# How might secondary dementia prevention programs work in practice: a pre-implementation study of the APPLE-Tree program

**DOI:** 10.1186/s12877-024-04762-3

**Published:** 2024-02-26

**Authors:** Rachel M. Morse, Iain Lang, Penny Rapaport, Michaela Poppe, Sarah Morgan-Trimmer, Claudia Cooper

**Affiliations:** 1 Centre for Psychiatry and Mental Health, Wolfson Institute of Population Health, Queen Mary University of London, London, UK; 2 Department of Health and Community Sciences, University of Exeter Medical School , Exeter, UK; 3 UK National Institute for Health and Care Research (NIHR) Applied Research Collaboration South West Peninsula (NIHR PenARC), Exeter , UK; 4 Division of Psychiatry, University College London, London, UK

**Keywords:** Dementia, Mild cognitive impairment, Subjective cognitive decline, Prevention, Implementation science

## Abstract

**Background:**

Over 850,000 people in the UK currently have dementia, and that number is expected to grow rapidly. One approach that may help slow or prevent this growth is personalized dementia prevention. For most people, this will involve targeted lifestyle changes. These approaches have shown promise in trials, but as of yet, the evidence for how to scale them to a population level is lacking. In this pre-implementation study, we aimed to explore stakeholder perspectives on developing system-readiness for dementia prevention programs. We focused on the APPLE-Tree program, one of several low-intensity, lifestyle-based dementia prevention interventions currently in clinical trials.

**Methods:**

We conducted semi-structured interviews with health and social care professionals without previous experience with the APPLE-Tree program, who had direct care or managerial experience in services for older adults with memory concerns, without a dementia diagnosis. We used the Consolidated Framework for Implementation Research to guide interviews and thematic analysis.

**Results:**

We interviewed 26 stakeholders: commissioners and service managers (*n* = 15) and frontline workers (*n* = 11) from eight NHS and 11 third sector organizations throughout England. We identified three main themes: (1) favorable beliefs in the effectiveness of dementia prevention programs in enhancing cognition and wellbeing and their potential to fill a service gap for people with memory concerns, (2) challenges related to funding and capacity to deliver such programs at organizations without staff capacity or higher prioritization of dementia services, and (3) modifications to delivery and guidance required for compatibility with organizations and patients.

**Conclusion:**

This study highlights likely challenges in scale-up if we are to make personalized dementia prevention widely available. This will only be possible with increased funding of dementia prevention activities; integrated care systems, with their focus on prevention, may enable this. Scale-up of dementia prevention programs will also require clear outlines of their core and adaptable components to fit funding, patient, and facilitator needs.

## Background

Dementia poses a significant health and societal challenge, with the estimated global number of people living with dementia projected to increase to 115 million by 2050 [1]. With evidence-based pharmacological approaches limited to management of vascular risk factors, current dementia prevention strategies focus on non-pharmacological, lifestyle-based approaches [2]. In 2020, the Lancet Commission on dementia outlined modifiable risk factors for dementia throughout the life course including: smoking, depression, social isolation, physical inactivity, diabetes, alcohol consumption, obesity, and hearing loss. Programs addressing these may prevent or delay up to one third of dementia cases [3].

Several randomized controlled trials have investigated multidimensional, lifestyle-based approaches to dementia prevention. The FINGER (Finnish Geriatric Intervention Study to Prevent Cognitive Impairment and Disability) trial investigated the use of a multi-component, two-year intervention with a focus on diet, exercise, cognitive training, and vascular risk factors (e.g., blood pressure) to prevent cognitive impairment in older adults at high risk for dementia. Ngandu et al. (2015) found a significant increase in cognitive function compared to the control [4]. Another trial, the Multidomain Alzheimer Preventive Trial, investigated a three-year, multi-component intervention with cognitive training, physical activity, nutrition advice, and omega-3 supplements to prevent cognitive decline in older adults with subjective memory complaints, slow gait speed, or difficulties with independent living. While initial analyses did not find any significant effects on cognition, Chhetri et al. (2021) found improved cognitive function in an at-risk subgroup who received the intervention and supplement [5]. The PreDiva (Prevention of Dementia by Intensive Vascular Care) trial evaluated a 6-year multi-component cardiovascular intervention for dementia prevention in cognitively healthy older adults. There was no significant change in incidence of dementia compared to the control [6].

These mixed results suggest that interventions targeting individuals at high risk of developing dementia or interventions that cover a wider range of lifestyle factors (as in the FINGER trial) may be most likely to benefit cognition. However, these interventions are intensive and difficult to scale to a population level. For example, delivery of the FINGER intervention is costly, requiring over 200-hours of expert time per participant. More vulnerable patient groups, including those at high risk of developing dementia, may be less likely to participate in time-intensive interventions like this, despite greater potential benefit [7].

Individuals at high risk of developing dementia include those with Subjective Cognitive Decline (SCD)– subjective impairment in the absence of objective impairment– or Mild Cognitive Impairment (MCI)– objective cognitive impairment exceeding what would be expected for one’s age, without significant daily impairment [8, 9]. SCD and MCI affect, respectively, 50% and 20% of adults over the age of 65 [10, 11]. In the UK, older adults presenting to primary care with SCD and MCI have a nearly 50% chance of receiving a diagnosis of dementia within three years [12].

The APPLE-Tree study (Active Prevention in People at risk of dementia: Lifestyle, bEhaviour change and Technology to REducE cognitive and functional decline) is a randomized controlled trial evaluating a wide ranging, lifestyle-based program’s effectiveness in reducing cognitive decline in older adults with SCD or MCI in England. APPLE-Tree does not require expert delivery, is less time-intensive than previous successful interventions, and is specifically tailored to people with SCD or MCI. If the APPLE-Tree program is proven to be clinically effective and cost-effective, it is intended to be scaled-up and implemented at National Health Service (NHS) and third sector sites throughout England.

In England, the current guidelines from the National Institute for Health and Care Excellence (NICE) and Public Health England promote dementia prevention [13, 14]; however, they primarily focus on risk factors related to cardiovascular disease (e.g., physical inactivity). There is limited reference to other modifiable, lifestyle-based risk factors for dementia including social isolation and mental health. Memory services are primarily commissioned to provide services to those with dementia; provision for people with SCD and MCI, beyond ruling out a dementia diagnosis, is usually very limited. People with SCD or MCI feel in a liminal state between health and dementia, left to monitor and manage their memory problems without support [15]. A review of the implementation of dementia prevention policies and strategies in England found that implementation of prevention programs is limited and inconsistent [16].

Pre-implementation research aims to streamline the process of implementing and scaling-up new initiatives by reducing the historically extensive interval between the discovery of evidence-based interventions and their practical application. This research facilitates a deeper understanding of implementation settings and their ability and readiness to deliver the intervention. Crucial factors include acceptability, appropriateness, feasibility, and fidelity to the intervention design [17, 18]. Existing studies on the implementation of dementia prevention programs, such as a content analysis of the FINGER intervention and a scoping review, highlight the need for tactics such as educational meetings, program champions, altered incentive structures, improved educational materials, increased coordination and collaboration, and enhanced access to resources [19, 20]. These studies, however, have limitations; the scoping review mainly draws from papers over a decade old, and both studies exclusively consider the perspectives of primary care practitioners.

### Aim

This study aimed to understand the perspectives of frontline staff members, service managers, and commissioners on the ability of NHS and third sector organizations in England to deliver secondary dementia prevention programs such as APPLE-Tree. Our goal was to learn about the organizational arrangements, tasks, and processes needed for the effective implementation of such programs. By identifying the ideal pre-implementation context, this research provides crucial groundwork for effective implementation that leads to appropriate and sustainable scale-up of dementia prevention programs.

## Methods

### Design

We interviewed participants from NHS primary and secondary care services and third sector organizations throughout England.

### Ethics

This study received ethical approval through the London-Camden and Kings Cross National Research Ethics Committees (20/LO/0034). The protocol is also registered (ISRCTN17325135) [7]. All participants provided written, informed consent.

### Participant selection

We used a sampling framework and purposively selected sites to incorporate different geographical and organizational contexts. Within sites, we sought to include diversity of roles and staff demographic characteristics, if possible. Eligible participants were front-line staff members, service managers, or commissioners working for NHS or third sectors sites who were involved in either making decisions about service provision for, or worked directly with, people with SCD or MCI. Participants were excluded from the study if they were involved in providing the APPLE-Tree program at a participating trial site.

We identified potential participants through existing contacts or publicly available information. Additional participants were identified through recommendations from interviewees (snowball sampling). Recruitment ceased when the research team observed repetition in the raw data and no new findings emerging.

### Procedures

In advance of the interviews, participants were sent the participant information sheet and the APPLE-Tree program booklet. At the start of each interview the researcher summarized the APPLE-Tree rationale, program, and process, including the following information: APPLE-Tree is a group program for six to seven people who meet for 10 group sessions. Each session is led by two facilitators who are non-clinical staff. The group meets every other week on Zoom for one hour to discuss a variety of evidence-based ways to make changes to lifestyle and behavior to help memory. On the weeks in between the main group sessions, there are 30-minute online tea breaks with the two facilitators, which are shorter and more informal to encourage peer support. In addition, participants have 20-minute goal-setting phone calls with one facilitator every two weeks to plan individual behavioral change. The topics covered in the program include: healthy diet, making social connections, looking after physical and mental health, coping with anxiety and memory problems, reducing alcohol consumption and smoking, and getting a good night’s sleep. Facilitators are not clinically trained but are trained and supervised by psychology or psychiatry clinicians (see protocol paper for further information [7]). Following this explanation, participants were given the opportunity to ask questions about the study or the APPLE-Tree program.

The interviews used a semi-structured interview guide based on the Consolidated Framework for Implementation Research (CFIR). The CFIR is a macrolevel, theoretical framework that can be used to guide pre-implementation research, highlighting similarities and differences between and across settings [21]. The CFIR includes five major domains– intervention characteristics, outer settings, inner settings, characteristics of the individuals involved, and the process of implementation– and includes 37 constructs within these major domains [21]. The topic guide was created with input from experts in implementation science and clinical care of people with memory concerns. The topic guide focused on (1) the participant’s role in their organization and the services provided to people with SCD, MCI, or dementia (as relevant), (2) reflections on previous programs delivered in their organization and dementia prevention programs such as APPLE-Tree, (3) staff, funding, support, and training needed to implement APPLE-Tree and similar programs, and (4) practicalities of implementing dementia prevention programs in the context of the organization. Questions varied by staff level (e.g., service manager questions focused more on staff and funding while frontline staff questions focused more on support and training).

The interviewer (RMM) was a researcher on the APPLE-Tree trial, with a background in implementation science research. The interviews took place between September 2022 through February 2023 and were conducted over Zoom or in a private area within the participant’s organization. The interviews were audio recorded and transcribed verbatim, then deidentified by RMM prior to analysis. Interviews lasted between 20 and 72 min (average 48 min).

### Data analysis

This study used an iterative approach to thematic analysis. We developed a codebook using the CFIR. Four researchers (RMM, CC, PR, IL) deductively and inductively coded up to four of the same interviews and added new codes to the CFIR-based codebook to best reflect the content of the interviews. Following this, one researcher (RMM) coded the remaining interviews using NVivo. Once all coding was complete, we grouped the codes together to identify emerging themes.

## Results

### Sample characteristics

We interviewed eight NHS workers from eight sites and 18 third sector workers from 11 sites. 15 participants were service managers or commissioners and 11 were front-line workers. The participant characteristics are summarized in Table 1.

**Table 1:** Characteristics of participants

Characteristics	N (%)
**Gender**	
Female	18 (69.2)
Male	8 (30.8)
**Professional role**	
*Commissioners / Service Managers*	15 (57.7)
*NHS*	
Commissioner	1 (3.8)
Service Lead	1 (3.8)
Service Manager	1 (3.8)
*Third sector*	
Chief Executive Officer	2 (7.7)
Chief Operating Officer	1 (3.8)
Service Lead	2 (7.7)
Service Manager	7 (26.9)
*Front-line Workers*	11 (42.3)
*NHS*	
Consultant Psychiatrist	1 (3.8)
General Practitioner	2 (7.7)
Memory Clinic Nurse	2 (7.7)
*Third sector*	
Support Worker	6 (23.1)
**Time working with people with memory concerns** ^**+**^	
≤2 years	8 (34.8)
3–5 years	5 (21.7)
6–10 years	3 (13.0)
10 + years	7 (30.4)
**Location of participant’s organization**	
Rural	3 (11.5)
Suburban	3 (11.5)
Urban	12 (46.2)
Mixed^*^	8 (30.8)

^***+***^ *N = 23– three participants did not provide this information*

^*^ *Refers to services with patients in a mix of urban, suburban, and rural areas*

Overall, we found three main themes. First, participants expressed positive views on the potential of dementia prevention programs to improve cognition and wellbeing, and their capacity to bridge a service gap for individuals with MCI or SCD. Second, participants discussed challenges related to funding and capacity to provide dementia prevention programs at sites lacking staff or where dementia services are prioritized. Third, participants discussed the need for adjustments in program delivery and guidelines to ensure compatibility with organizations and patients.

### Perceptions and potential benefits of dementia prevention programs

This theme reflects a belief that dementia prevention programs such as APPLE-Tree could improve cognition and wellbeing and that this capability addresses a gap in services for individuals with SCD or MCI.

Most participants (19/26) discussed issues that fit with the CFIR construct ‘evidence strength and quality’, describing APPLE-Tree and similar dementia prevention programs as capable of positively influencing cognition. When asked whether a lifestyle-based prevention program can influence cognition, one participant stated, “*Definitely. They’re all evidence-based, and the exercise, eating well, everything that keeps us healthy physically also is good for your brain*,” (third sector service manager, #3). Another stated:

*Absolutely. And I think it’s information that is coming more and more to light now about especially physical activity and keeping your brain active and all those other things that I don’t think people really think about still… I think it will be an eye opener for people, I think they still don’t realize that all of these things can have this impact on cognitive decline* (third sector service manager, #11).

A minority (5/26) of participants were uncertain about whether APPLE-Tree and similar dementia prevention programs could influence cognition. One participant stated that the topics in the APPLE-Tree program were, “*stuff that can maybe not make the memory loss any better*,” (third sector front-line worker, #9). However, these participants also expressed a desire for published evidence demonstrating the effects of APPLE-Tree: “*I’m not convinced, but I’ll happily be convinced that it has an impact on cognition, and but I think absolutely it’s something that is required and something that should be encouraged,”* (NHS service manager, #16). Another participant stated, *“If the evidence shows that there is benefit, then by all means, we should pursue that,”* (NHS front-line worker, #22).

While some participants were uncertain about whether dementia-prevention programs affected cognition, nearly all participants (25/26) spoke positively about the programs’ ability to improve wellbeing, quality of life, and general health (CFIR construct: ‘knowledge and beliefs’). One participant stated:

*I just know it will have benefit to our clients, I just know it will have benefit to the population, I just know it will. Anything like this, because you’re talking about improving people’s health, not just mental health but emotional and physical health as well and that’s going to have a big positive impact* (third sector service manager, #13).

Another participant described their positive belief about the programs’ ability to improve general health, including risk factors for dementia:

*I think it will also help, not just with their memory, but will help with quite a lot of different aspects of their lives as well. It will probably help with their blood pressure and the cholesterol and all the other things that they’re going to be checked for anyway, through the GP. So it’s good for like just general health and wellbeing* (NHS frontline worker, #15).

Many participants also identified a gap in the services available for people with SCD or MCI but without a dementia diagnosis. Within the CFIR constructs ‘tension for change’ and ‘compatibility’, participants described APPLE-Tree as a program that could fill this gap. Only two sites (one NHS, one third sector) out of 19 involved in this study had services available specifically for people with MCI, and none had services specifically for people with SCD. One participant described the lack of services available to people with MCI: “*I can certainly see that this group of people that are getting diagnosed with mild cognitive impairment, where they have nothing really tailored to their needs and their situation,”* (third sector service manager, #23). Another stated:

*People didn’t know what it [MCI] was… they didn’t get any information at all on it… So they didn’t know what to expect. They were scared. They didn’t know whether it was going to mean they were going to get dementia or not, so there was just nothing, really, and no support* (third sector service manager, #3).

Many of these participants subsequently described dementia prevention programs, including APPLE-Tree, as filling this gap in the services. One participant stated: “*If it’s MCI generally they’re just, kind of, discharged back to the GP and nothing much happens to them at the moment so I’m hoping that’s where APPLE Tree will fit in.”* (NHS commissioner, #20). Another participant said:

*That group of people [with MCI] tend to get forgotten about, and I think it’s absolutely brilliant that there could be something that people could do, something practical that they could do that could, one, help facilitate them potentially not– their memory not declining, but also enabling them to meet other people and talk to other people in a similar situation* (third sector service manager, #23).

### Funding, priority, and sustainability

This theme focuses on two related topics: the priority given by sites to dementia-prevention programs such as APPLE-Tree, and the challenges around resourcing the sustainable implementation of such programs.

Some participants were enthusiastic about the importance and likelihood of their site funding such a program through applications for additional funding from commissioners or local councils; others were not. Within the CFIR construct ‘relative priority’, this divide was discussed as due to a lack of staff capacity to deliver programs like APPLE-Tree or due to the focus of the organization (e.g., a focus on dementia only). Interviewees from eight out of 11 third sector sites supported finding funding. Interviewees from three third sector sites said their site would likely not support finding funding; two of those sites focus specifically on dementia and one on homelessness. In each case, participants said that the target population of APPLE-Tree was not part of their sites’ remit. Four out of eight NHS sites supported finding funding with the rest stating they were at capacity. One NHS service manager stated, “*I just think that we need to think about how it’s funded, and who manages it, and who provides it because it can’t happen as an extra thing within the services that I provide because I just wouldn’t be able to then do the 150, 160 dementia diagnoses every month, you know?”* (NHS service manager, #16).

Fifteen (out of 26) participants mentioned a lack of resources, specifically a lack of staff, available to deliver programs like APPLE-Tree (CFIR construct: ‘available resources’). This response was raised much more often at NHS rather than third-sector sites. One participant stated: “*We’re all hugely stretched and understaffed*,” (NHS commissioner, #20). Another described, “*They’ve got 300 people on their caseload. They’re supposed to be doing one visit each month, all unrealistic because we’re human, we react to emotions and it’s not happening. So they’re kind of like really, really overworked*,” (NHS frontline worker, #18).

A minority of participants (7/26) considered dementia prevention programs to be a low priority within their services. Instead, they said funding and resources should be prioritized for services specifically targeting people already diagnosed with dementia. These responses tended to come from participants at dementia-focused third-sector sites and at NHS sites. For example, one participant described APPLE-Tree’s priority in their service in this way:

*I think it’s [APPLE-Tree] fantastic and my only concern would be if we roll that out nationally what the cost is in a service that’s hugely over stretched. So, you know, you’re giving this gold-plated Rolls Royce service, which is wonderful, and yet I’ve got patients who have actually got a diagnosis of dementia who can’t even have a once-a-year hospital follow up* (NHS commissioner, #20).

Another said, “*We have to deliver an intervention to people with dementia first, and then we can deliver an intervention to people with MCI, or who don’t have a diagnosis*,” (NHS front-line worker, #22).

A greater focus on programs for people with dementia was seen among some participants (7/26) who mistakenly presumed the target population of APPLE-Tree was people with dementia, despite the summary of the intervention at the start of the interview. One participant stated, “*So providing goal settings, wellbeing support, activities tailored to supporting people with various diagnoses, specifically dementia, it’s really important because it helps with coming to terms with the diagnosis,”* (third sector frontline worker, #6). Another stated, “*your study is dominantly for the person with a dementia*,” (NHS service manager, #19).

Participants who supported finding funding for APPLE-Tree and similar programs commonly discussed the need to apply for additional funding from commissioners or the local council. Within the CFIR construct ‘cost’, one participant described, “*We get a lot through grants from the councils, but we can also apply for funding,”* (third sector service manager, #8).

A minority of (5/26) participants, all from third sector sites, mentioned the possibility of funding dementia prevention programs by charging patients for each session: “*I mean, we have goals for how to make it a sustainable group, for instance, we do have to charge so much money for people to come along each week, to help make the service sustainable for us and not depend on funding*,” (third sector service manager, #12). Others thought it would be possible to find additional funding without charging patients. When asked about whether funding could be found, one commissioner responded, “*Absolutely. And, of course, it should be the case that you receive the funding just because you’re improving people’s quality of life*,” (NHS commissioner, #20).

Some participants were concerned about whether they would be able to find enough additional funding to deliver APPLE-Tree in its current form. One participant discussed the challenge of paying for a clinical psychologist to monitor the program: “*I hadn’t realized you needed it to be supervised by clinical psychologists, because again, you’re adding a cost to all this, and I’m not clear why that’s necessary, because obviously that is a very expensive cost,”* (third sector service manager, #3). Another participant described challenges with the program structure: “*It’s funding and if I can deliver, I suppose if it means that we can deliver ten sessions, for example, with one facilitator but could only deliver five with two, I might opt for the ten so that people are getting the most out of it as possible,”* (third sector service manager, #7).

While there were participants who thought they would be able to find at least partial funding for APPLE-Tree or similar programs, some thought this financial support was only likely to be temporary. One stated: “*When you apply for funding, you know, you get that short period of time but then they won’t continue your funding; they’re always talking to you about what your exit strategy is or how you’re going to match funds and things go out of favor like that, they always want something new*,” (third sector service manager, #1).

Participants proposed networking and collaboration with external organizations to improve sustainability. Nearly all (16/19) participating sites had a high degree of networking and collaboration and were already linked with other NHS or third sector sites (CFIR domain: ‘cosmopolitanism’). One participant described the possibilities for collaboration in this way:

*If you were running it in that way, you’d look about who in the community would want to facilitate that because they’ve got a particular interest in doing that session and involve people, in that kind of way, to do it. It might be that a local café says, “I’ll run that for you because we could run the smoothie-making in our kitchen,” and do you see what I mean? And then it becomes more sustainable rather than it being funded. It’s thinking in different ways because when you rely on commissioners to fund you for something, it’s flavor of the month for a year and after a year they go “no.”* (third sector service manager, #1).

Another participant outlined the advantages of collaborative approaches:

*There’s no reason why APPLE-Tree couldn’t get a group of likeminded sector organizations and do a collaborative bid. So we don’t have to do these bids on our own, if there was a group of organizations. And to be honest, a lot of funders today like to see collaborative bids, particularly if they extend the reach* (third sector service manager, #14).

Even participants who said their site had no capacity to deliver programs like APPLE-Tree discussed networking and collaboration with external organizations to refer individuals to sites that could provide such programs. One participant stated: “*We would refer on. I don’t think there’s any capacity to do [APPLE-Tree]*,” (NHS frontline worker, #15) while another stated, “*I think we could refer people to you. I don’t think we have the resources or capacity to actually deliver a group for you*,” (third sector service manager, #24).

### Dementia prevention program delivery and guidance

This theme discusses adjustments needed to make dementia prevention programs like APPLE-Tree compatible with organizations and patients, including a hybrid or face-to-face delivery and cultural adjustments, and discusses guidance needed for facilitators and patients.

Within the CFIR construct ‘complexity’, nearly all (25/26) participants expressed concerns about delivering APPLE-Tree and similar programs online. Many suggested a hybrid or face-to-face approach instead:

*I think that [Zoom] would make it less accessible for some people. I also think that it defeats the object a little bit, by having it online all the time. I’m not saying there’s no place for hybrid, because I absolutely think there is, but… I don’t know how people would effectively engage with each other over Zoom and socialize* (third sector service manager, #4).

Another participant described a similar worry:

*The only thing that I have a concern about is anything that’s online… it’s challenging delivering things online. Delivering face to face is a lot easier, and with our staff, they’d find delivering face to face a lot easier* (third sector service manager, #11).

Most (22/26) participants discussed changes needed to be made to APPLE-Tree to fit their patients’ needs (CFIR constructs: ‘adaptability’ and ‘patient needs and resources’). One example was delivering APPLE-Tree in languages appropriate to their patients: “*We’ve got a high proportion of people that are from Turkish origin and the language is a massive barrier… so immediately, I can think there’ll be an issue about translation*,” (third sector service manager, #1). Another example related to the affordability of foods discussed in the program: “*I worry about how people can afford that [the foods], and the affordability of doing that each week*,” (NHS frontline worker, #17). Other participants mentioned a need to adjust the recipes to be culturally appropriate: “*So we’ve got to make it culturally appropriate. We’ve got to make our dietary advice compatible with the person’s lifestyle and, as we were saying before, their financial resources*,” (NHS commissioner, #20).

Many (11/26) participants discussed guidance from APPLE-Tree and similar dementia prevention programs that would be useful in the implementation process. Some participants asked for guidance for their facilitators in delivering APPLE-Tree:

*I’d take the guidance from you really in terms of what skillset do you need and what would be the commitment, obviously how much would you want to pay the facilitators, although most people wouldn’t do that because of the money, you know, it’s mostly time and expense isn’t it. And how would they receive the training, would it involve them having to travel to London or would the training be done online, that kind of thing* (third sector service manager, #8).

Another participant said that peer support and training for facilitators would be useful:

*Yes, like a group of other facilitators who can bounce off each other as well, I think that would be helpful. Other than that, and making sure you’ve had all the right training to get up and start it, I can’t see that there would be any big issues in getting it going other than funding* (third sector service manager, #12).

Other participants described the need for additional resources for participants to be able to refer to after the program: “*I wonder if there is a need for something a little bit after, after care, yeah, even if it is just a website or something to ask questions*,” (third sector service manager, #7).

## Discussion

Across NHS and third sector sites, we found favorable beliefs about the ability of group secondary prevention programs such as APPLE-Tree to improve cognition and wellbeing. These beliefs fit within the CFIR constructs ‘evidence strength and quality’ and ‘knowledge and beliefs’. Our findings are consistent with previous research on the perspectives of primary care providers regarding dementia risk reduction, which found that the majority of providers view dementia risk reduction as achievable [22, 23]. Participants wanted to see evidence that APPLE-Tree and similar programs work before they would agree to implement them, which highlights the importance of establishing and communicating the effectiveness of programs of this type. Robust data on clinical effectiveness is crucial in this process; it is a key component of knowledge translation and exchange and facilitates the adoption and sustainable implementation of these programs [ 24 ].

There was a lack of services available for people with SCD and MCI at sites included in our study; only two of the 19 study sites had resources specifically for people with MCI and none had resources tailored to people with SCD. This is in line with previous findings that older adults with SCD and MCI in England often lack access to memory services and feel left to manage their memory issues on their own [15]. Our participants identified APPLE-Tree and similar programs as having potential to fill this gap, describing an implementation context in which there is both a need to change the current system and a potential for programs like APPLE-Tree to facilitate the change.

While our participants regarded dementia prevention programs such as APPLE-Tree as valuable and as addressing a gap in services, they were divided over the issue of funding; some supported applying for funding and some did not. Among the third-sector sites that did not support applying for funding, the primary reason was that dementia prevention did not fall within their site’s scope. For the NHS sites that did not support applying for funding, the reasons stated included a lack of staff time and capacity, as well as a need to prioritize services for individuals with dementia. A study on barriers and facilitators to implementing the FINGER intervention also found a lack of staff time and a focus on providing dementia services, rather than preventative services, to be barriers [20]. Similarly, the scoping review on implementing dementia risk reduction interventions in primary care found insufficient availability of staff to be a barrier [19].

Given the well-documented strains on health and social care services in England, it was unsurprising that some participants concluded that funding for dementia prevention programs, such as APPLE-Tree, is unfeasible or not a priority [16, 25]. Additionally, for many participants, the limited resources that currently fund their existing, care-focused work could also not be stretched to cover dementia prevention and dementia care services. It is notable that even with explanation at the start of the interview, several participants assumed the intervention was for dementia care, perhaps indicating that dementia prevention is not currently a salient concept or prioritized in current NHS services. This highlights the extent of policy shift that the first scalable, clinically effective and cost effective non-pharmacological dementia prevention programs will require. With the cost of dementia care in the UK expected to rise from £34.7 billion in 2019 to £94.1 billion in 2040 [26], preventing dementia is not only valuable for individuals and society but also potentially cost-effective. A NICE technology evaluation of the first lifestyle-based dementia prevention programs might well find these approaches more cost-effective than expensive dementia treatment drugs (e.g., Lecanemab). Such an evaluation could substantially increase the allocation of resources for dementia prevention, underscoring the critical role of cost-effectiveness data in shaping health policies and facilitating the implementation of dementia prevention programs. These programs will be essential if UK health policy is to deliver on plans for the 2020s as the decade of proactive personalized prevention [27].

Services aiming to meet these personalized prevention goals through implementation of dementia prevention programs will need adequate resourcing. The ability of APPLE-Tree to be delivered by a non-clinical workforce will make this more attainable. However, in the absence of a national level policy shift, APPLE-Tree scale-up may not be feasible at overstretched NHS sites or third sector sites with a specific focus on dementia. Several participants considered such interventions would be valuable but needed to sit apart from dementia services. This may also be the choice of future clients too; in co-design, stakeholders with lived experience of memory concerns advocated against references to dementia in the APPLE-Tree intervention, situating it instead as a cognitive wellbeing course. They felt this approach reduced potential fear and stigma and also avoided an implication that prevention, as opposed to risk reduction could be guaranteed on a personal level [28].

In England, the 2022 Health and Care Act introduced integrated care systems (ICSs), which involve collaborations among organizations to plan and deliver integrated health and care services. One component of ICSs, integrated care partnerships, aims to improve outcomes in population health and health care through statutory committees that bring together a broad set of system partners (including local government, the voluntary, community, and social enterprise sector, NHS organizations, and others) to develop a health and care strategy for the area [29]. Through shared responsibility for budgets and outcomes, they present an opportunity to take a whole system approach to prevention and are potentially well placed to consider where interventions such as APPLE-Tree are best deployed.

Reflecting concerns about how a dementia prevention program might be introduced without compromising current services, participants suggested trade-offs to deliver more with less: reducing the number of facilitators and number of program sessions. This is a critical challenge in implementation science; there is often tension between the need to adapt a program to a given context, in this case a hypothesized funding envelope, and the need for program fidelity (i.e., adherence to core components of the program) [30]. One method of addressing this tension is through using the Dynamic Adaptation Process (DAP). The DAP includes identifying the fundamental components and adaptable traits of a program. This would then be followed by providing instruction on permissible modifications to the program and supporting implementation of the modified program through monitoring and evaluation [31].

In both NHS and third sector settings, APPLE-Tree and similar programs may need to be adapted for different patient populations. Trial populations, despite the best efforts of researchers, are never wholly representative of populations for whom interventions are intended, though more so for non-pharmacological trials compared with drug trials [32]. Participants in this study discussed needing to adapt APPLE-Tree to technological capabilities, cultural differences, and financial needs to reach and engage target populations. These adaptations can be considered using the DAP. For example, this would allow consideration of whether delivery of APPLE-Tree online constitutes a core or adaptable component. However, there are also frameworks more specific to cultural adaptation, including the Ecological Validity Model [33] or the Cultural Adaptation Process. The Cultural Adaptation Process begins with collaboration between stakeholders to evaluate patient needs and continues with adaptations to the program, pilot testing, and trialing with constructive feedback provided throughout the process [34]. This enables organizations to tailor dementia prevention programs to address specific considerations, such as the financial constraints of their patient population when recommending changes to their diet. Moreover, it provides an opportunity for conducting further research in settings beyond the UK to facilitate exploration of adaptations needed for broader patient populations. In doing so, this research can help reduce disparities in access to dementia prevention programs, ensuring that these programs are available to patients of varied socioeconomic status or cultural circumstances.

Provision of supplementary materials to participants and facilitators (e.g., access to a website with nutritional information) could support adaptability. An implementation process that defines these considerations as core or adaptable components will help to provide sufficient guidance without affecting fidelity to the program.

### Limitations

This is a pre-implementation study; assessment of the clinical effectiveness and cost-effectiveness of APPLE-Tree are in progress but have not been completed. This study considers stakeholders’ perspectives on a lifestyle-based approach to secondary dementia prevention. With proof of concept from existing trials that such approaches can reduce cognitive decline, there is reason to be optimistic that scalable versions, whether from APPLE-Tree or other similar programs, will emerge in the next decade. In advance of this, pre-implementation work such as this may decrease the time required to implement such programs. We do not consider the health economics of implementation; however, interviewees highlighted importance of funding and economics as key drivers of implementation. Given the value of previous examination of the health economics of the first dementia treatments [35], a similar analysis for dementia prevention would be valuable to policymakers [36, 37]. Our findings are based on research done in NHS and third sector settings in England and may not be generalizable to other locations. However, they provide valuable information on implementation of dementia prevention programs in the NHS and more broadly at third sector organizations.

## Conclusion

This study explored the optimal pre-implementation context for scaling-up lifestyle-based dementia prevention programs including APPLE-Tree. We use the Consolidated Framework for Implementation Research to outline the arrangements necessary for sustainable implementation. Participants described favorable beliefs about how dementia prevention programs could positively impact cognition and wellbeing and about their ability to fill the existing gap in services for people with MCI or SCD. Challenges in implementing these programs may arise due to sites without staff capacity or with a higher prioritization of dementia services. Sites delivering dementia prevention programs would benefit from a concise outline of core and adaptable program components to meet funding, patient, and facilitator needs. By utilizing health economic data to drive evidence-based policies and investment in dementia prevention, there is an opportunity to influence national policy towards funding cost-effective dementia prevention programs resulting in potential for savings from reducing dementia prevalence.

## Data Availability

Data and materials are available on reasonable request to the corresponding author.

## Abbreviations

APPLE-Tree: Active Prevention in People at risk of dementia: Lifestyle, bEhaviour change and Technology to REducE cognitive and functional decline
FINGER: Finnish Geriatric Intervention Study to Prevent Cognitive Impairment and Disability
PreDiva: Prevention of Dementia by Intensive Vascular Care
SCD: Subjective cognitive decline
MCI: Mild cognitive impairment
NHS: National Health Service
NICE: National Institute for Health and Care Excellence
CFIR: Consolidated Framework for Implementation Research
ICS: Integrated care systems
DAP: Dynamic Adaptation Process
